# Application of Polymers in Hydraulic Fracturing Fluids: A Review

**DOI:** 10.3390/polym17182562

**Published:** 2025-09-22

**Authors:** Amro Othman, Murtada Saleh Aljawad, Rajendra Kalgaonkar, Muhammad Shahzad Kamal

**Affiliations:** 1 Center for Integrative Petroleum Research, College of Petroleum Engineering and Geosciences, King Fahd University of Petroleum and Minerals, Dhahran 31261, Saudi Arabia; amro.othman@kfupm.edu.sa; 2 Petroleum Engineering Department, College of Petroleum Engineering and Geosciences, King Fahd University of Petroleum and Minerals, Dhahran 31261, Saudi Arabia; 3 EXPECR ARC, Saudi Aramco, Dhahran 31311, Saudi Arabia; rajendra.kalgaonkar@aramco.com

**Keywords:** polymers, fracturing fluids, environmental impact, formation damage, hydration, crosslinking, breaking

## Abstract

Multistage hydraulic fracturing significantly increased oil and gas production in the past two decades. After drilling, fracturing fluids are pumped into the formation to create fractures that provide pathways to the hydrocarbon. These fluids are usually viscous to provide the mechanical power to frack the formation and carry the proppants, which keep the fractures open. After fracking, the viscous gel should be broken to allow the flowback of the fluid to avoid formation damage. The key player in the fracturing fluid system is the polymer, which is responsible for the fluid viscosity of the system. All other additives are added to improve the polymer’s performance under different conditions and reduce formation damage. The formation damage appears as fine migration, residue precipitation, adsorption, and wettability alteration. All of these types are affected by the polymer types and behavior. This paper reviews the polymers used in fracturing treatments, their classifications, preparations, mechanisms, degradation behavior, and interactions with other fracturing fluid additives. It also covers their impact on the formation damage and environmental concerns raised with fracturing treatments, including spills and flaring activities. The paper discussed the cost of the main polymers used in fracturing fluids and suggested practical recommendations to select a robust, cost-effective polymer. By integrating these concepts, the review gives the researcher the necessary knowledge to design and prepare effective fracturing fluids tailored to a wide range of operational scenarios.

## 1. Introduction

Polymers are extensively used in the oil and gas industry to enhance fluid performance. They can be chemically modified or combined with additives, such as cross-linkers and breakers, to enhance their effectiveness. They are used to control the viscosity, minimize fluid loss, and facilitate efficient material transport within the reservoir. The polymers are widely used across various oil and gas applications, including upstream applications such as drilling, enhanced oil recovery (EOR), water shut-off, and hydraulic fracturing. In drilling fluids, polymers are used to enhance cuttings transport and reduce fluid loss in water-based muds. The polymers used in drilling are classified as natural, such as xanthan gum, guar gum, starch, and cellulose derivatives, or synthetic, including polyacrylamide/hydrolyzed polyacrylamide (PAM/HPAM), polyacrylates, and 2-acrylamido-2-methylpropane sulfonic acid, AMPS-based copolymers. These polymers are used in water-based drilling fluids [1]. Xanthan provides high viscosity but is heat-sensitive; guar controls viscosity but is prone to bacterial and thermal breakdown; starch limits fluid loss but lacks heat resistance. Carboxymethyl cellulose (CMC) and polyanionic cellulose (PAC) control filtration but degrade under harsh conditions [2]. PAM is used to build viscosity, control filtration, and stabilize shales, but it loses effectiveness in high salinity, high temperature, and high shear. Nanopolymer additives improve thermal and salt tolerance, reduce filtration, and strengthen wellbore stability, yet environmental and limits in extreme conditions remain [1]. Enhanced oil recovery uses polymers to improve mobility control and increase oil displacement. Both natural and synthetic polymers are used. Natural polymers such as xanthan, guar, and cellulose derivatives are environmentally friendly and provide salinity tolerance and thickening; however, when thermal stability is critical, guar and cellulose derivatives are preferred [3,4,5,6]. Xanthan is prone to microbial breakdown and costs more than common synthetic polymers, which limits its use in EOR. It also loses viscosity at high temperatures, reducing effectiveness in harsh reservoirs and highlighting the need for more thermally stable, cost-effective biopolymer alternatives [7]. Synthetic polymers used for mobility control, including PAM, HPAM, and hydrophobically associating polyacrylamide (HAPAM), have spanned broad product-dependent molecular-weight ranges. The HPAM is widespread, sensitive to temperature and shear stress, and is not biodegradable [8]. The performance of HAPAM, Xanthan and guar was compared directly in a side-by-side test at 1000, 4000, and 6000 ppm at 30 °C, HAPAM has been shown to maintain higher viscosity at high shear and to deliver greater cumulative oil recovery (41.1–63.5%) than xanthan (32.8–56.2%) and guar (41.8–61.2%). The least permeability impairment has been observed with HAPAM, about 28% versus about 54% for xanthan and about 35% for guar. By contrast, the effectiveness of HPAM has been reduced in hot, saline brines because hydrolysis and precipitation with divalent cations have been promoted. Accordingly, under the studied conditions, HAPAM has been identified as the superior mobility-control agent [9].

Chemical gels provide a cost-effective solution for water shut-off applications than mechanical methods. These gels are composed of polymers, which dominate the industry, with nanosilica-based formulations emerging. An optimized polymer concentration can enhance oil production while lowering water cut during treatment. Cross-linked polymer systems have demonstrated promising performance in these applications, though precise zone targeting is essential to avoid blocking oil zones. The most used materials in water blocking are PAM and organic cross-linkers, such as polyethylenimine (PEI). They can offer long-term control over water production [10,11,12]. The combination of PAM and PEI forms stable hydrogels, which are widely used worldwide to address water management issues like water coning and high-permeability zones [13]. However, they have limitations in thermal stability. They become less effective in reservoirs above 80 °C [11]. Numerous modifications have been made to this system to improve polymer performance. PAM-based copolymers and organic crosslinkers were introduced into the water shut-off field [14]. HPAM and cross-linkers can block the water zone and reduce the water cut. El Karsani et al. (2015) reported that the HPAM/PEI possesses excellent mechanical strength and thermal stability at temperatures up to approximately 130 °C [15]. Additionally, by incorporating an emulsifier, the system evolved into an emulsified crosslinked polymer that can be injected without the risk of prematurely blocking oil zones, as the emulsion delays crosslinking and directs the polymer toward water-producing zones [16].

### 1.1. Hydraulic Fracturing

The success of any fracturing operation largely depends on the design of the fracturing fluid, which must meet specific performance criteria such as viscosity, thermal stability, and formation compatibility. A wide variety of fracturing fluids are used, including water-based, oil-based, energized, and hybrid systems, each tailored to suit specific reservoir conditions. To optimize the performance of these fluids, various chemical additives are added. The following part discusses hydraulic fracturing operations, additives, and a brief introduction to polymer types used in fracturing treatments. The hydraulic fracturing technique has existed for decades, but it has recently become essential to production when combined with horizontal drilling. This method is widely used in large-scale treatments to improve fluid flow in unconventional formations. Fracture design depends on factors such as rock type, reservoir conditions, and freshwater availability within budget limits. Key elements include fluid type, proppant, injection rate, and treatment volume [17]. The effective fracturing fluids open the fractures and carry the proppants, which keep the fractures open, to allow the easy flow of hydrocarbons. These fluids are designed to minimize pressure loss, reduce fluid leak-off, ensure ease of break and flow back, minimize formation damage, and remain compatible with formation fluids [18,19]. The first hydraulic fracturing treatment in 1947 used gelled crude oil, kerosene, and sand. Over time, water-based fluids with surfactants and clay stabilizers became standard. The technique evolved with large-scale operations in the 1960s, cross-linking agents were introduced in the 1970s, and horizontal drilling in the 1980s. By 2012, more than 2.5 million treatments had been performed worldwide [20,21]. By 2016, 69% of all oil and natural gas wells drilled in the U.S. were fractured horizontal wells [22]. In hydraulic fracturing treatments, high-pressure fluids are injected to create fractures in the reservoir formations.

### 1.2. Fracturing Fluids

Fracturing fluids are either (a) *Oil-based fracturing fluids*, which are low water usage and better fluid handling, and also have the slowest proppant settlement and highest fracture conductivity [23,24]. (b) *Energized/foam fracturing fluids* also minimize water usage, reducing clay damage and improving hydrocarbon recovery by gas expansion near the fracture. Common foam systems include CO_2_ and N_2_ with hydroxypropyl guar (HPG), and foaming agents are used with slickwater, cross-linkers, and viscoelastic surfactants to reduce formation damage [24,25]. (c) *Water-based fracturing fluids* face high costs, water consumption, formation damage, and disposal issues. Common types include high viscosity gelled fluids, which are guar and their derivatives with cross-linkers to increase viscosity and proppant transport efficiency, requiring breakers for flow back. Slickwater contains over 90% water with small amounts of friction reducers or guar derivatives, requiring high pump rates and causing potential emulsion, water blockage, and scaling issues. Viscoelastic Surfactants prevent polymer residue damage by using surfactants to form micelles, increasing viscosity without cross-linkers, and breaking down upon oil or gas contact. Alcohol and Emulsion fluids are specialized fluids for specific conditions. Hybrid fluids combine slickwater and cross-linked fluids to optimize fluid properties [25].

The selection of the additives used in fracturing fluids is influenced by reservoir temperature, water chemistry, and economic factors. These additives are used to enhance thermal stability, regulate pH, control fluid loss, and minimize formation damage. Understanding their interactions under reservoir conditions is essential for designing fracturing treatments and achieving optimal performance [26]. Gelling agents, mainly polymers, are the essential components of fracturing fluids; all other additives are to support and enhance the polymer performance. At the early stages of fracturing treatments, the polymers are used to prepare linear gels, named the pre-pad. The pre-pad fluid is used to condition the formation and initiate the fractures. In the second stage, the polymers are attached to a specific group to be cross-linked and named a pad. The pad is used to initiate and propagate the fracture, and then the proppant is carried in the pad fluid, and the fluid is named slurry at this stage [24,27]. Cross-linkers, including borate salts, enhance fluid elasticity and viscosity instantly without increasing polymer loading, whereas delayed cross-linkers such as titanium, and zirconium oxychloride, improve proppant transport [28,29,30,31]. Breakers like bromates, peroxy disulfate, and enzymes reduce viscosity after fracture closure to release proppant, though they pose a risk of premature screen-out, requiring controlled breakage to minimize damage [28,32,33,34].

pH buffers such as sodium hydroxide and potassium hydroxide regulate fluid pH to optimize polymer hydration and cross-linker efficiency for both linear and cross-linked gels. Bactericides, including glutaraldehyde and aldehydes, prevent bacterial growth and fluid degradation, which is critical for both freshwater and seawater-based fluids, as bacteria can release H_2_S [35]. Scale inhibitors like acrylic acid polymers and phosphates prevent scale formation during fracturing, with phosphonate gels controlling calcium scale at high temperatures, making them essential for seawater-based fluids [28]. Clay stabilizers such as tetra-methyl ammonium chloride and potassium chloride prevent clay swelling and migration, maintaining formation permeability, particularly in brine carrier fluids [36]. Surfactants, methanol, and isopropanol reduce interfacial tension, act as demulsifiers, and improve fluid recovery and flowback [37]. Hydrochloric acid and muriatic acid are used in 5–15% concentrations to clean cement, dissolve minerals, and initiate fissures before fracturing. Friction reducers like sodium acrylate, acrylamide polymer, and polyacrylamide optimize pump rate and pressure, improving efficiency in slickwater fracturing [38]. Chelating agents such as ethylenediaminetetraacetic acid (EDTA) and L-glutamic acid, *N*,*N*-diacetic acid (GLDA) capture ions that damage formations. The GLDA is effective up to 300 °F, and reduces interfacial tension and controls reaction rates in high-temperature seawater treatments [39]. Proppants, including natural sand, ceramic, and resin-coated proppants, are selected based on strength, density, and sphericity to keep fractures open after fluid breakdown and ensure optimal conductivity [40].

This review explores the primary polymers used in hydraulic fracturing, emphasizing their classification, preparation methods, functional roles, and interactions with various additives. It details the key mechanisms involved in fracturing fluid formulation, including polymer hydration, cross-linking, and breaking processes. With the growing complexity of hydraulic fracturing environments—ranging from ultra-deep wells to high-temperature, high-salinity formations—the question arises: can these traditional polymers continue to meet the escalating demands of modern operations, or are new, more resilient, and complex alternatives required? The review also highlights the influence of temperature, salinity, and pH conditions in optimizing fluid performance at different stages of the operation. Numerous studies are referenced throughout to support the discussion. Additionally, the paper addresses common challenges such as formation damage, environmental impact, and cost considerations. Lastly, it presents recommendations and prospects, including alternative polymers that remain underutilized but offer enhanced performance and reduced environmental impact.

## 2. Polymer Used for Hydraulic Fracturing

Polymers are versatile materials classified based on their origin, structure, behavior in solution, functional properties, and thermal behavior. Understanding these classifications aids in selecting the appropriate polymer for applications such as hydraulic fracturing. The following part classifies the polymers for hydraulic fracturing based on many criteria.

### 2.1. Classification Based on Origin and Source

- Natural polymers come from natural sources like plants and animals. Examples include guar gum, cellulose such as CMC, and microbials such as Xanthan.
- Synthetic polymers are synthesized through chemical processes. Examples include polyacrylamide (PAM) and derivatives.
- Semi-synthetic polymers are modified natural polymers, such as cellulose acetate or rayon, which are chemically treated to enhance their properties [41].

Table 1 provides a detailed classification based on origin and source, including examples. The polymers listed are specifically those used for hydraulic fracturing and other applications in the oil industry.

### 2.2. Other Classification

There are many other classifications to classify the polymers, including: based on polymer structure, which is either linear polymers, branched polymers, or cross-linked polymers that have a 3D network [41]. The polymers are also classified based on the presence of electrical charges within their chains to non-ionic, ionic, amphoteric (contains acidic and basic groups), and zwitterionic (contains anionic and cationic groups) [47]. Based on behavior in water, the polymers can be grouped into hydrophilic polymers (water soluble), such as guar gum, and hydrophobic (do not dissolve in water) [48]. The functional properties are used to measure the performance of polymers, such as rheological properties and interfacial effects. They divide the polymers based on behaviors into structural polymers, adhesive polymers, and functional polymers. The functional polymers exhibit specific chemical or biological functions, such as guar gum, which is used for its thickening and gelling properties in various industries [41]. The thermal properties indicate how a polymer responds to temperature. The first group of polymers under thermal properties classification is thermoplastics, which include amorphous and crystalline. The second group is thermosets, while the third group is the elastomers, which include thermoset elastomers and thermoplastic elastomers. Thermal classification of polymers is based on their response to heating and cooling cycles: thermoplastics can be reshaped with heat, thermosets harden permanently, and elastomers are elastic and may belong to either group [49,50,51]. These classifications determine the suitable polymer for the suitable job and operating conditions, i.e., the structure and charge determine the compatibility of the polymer with the formation brine. The functional properties determine the job based on its needed rheological properties. The thermal properties determine the effective window for the polymer. These kinds of classifications and properties can help to select the right polymer for specific formation, operating conditions and available resources.

The following section reviews the key polymers used in hydraulic fracturing treatments, guar derivatives, CMC, and xanthan gum, focusing on their preparation, chemical modification, properties, and practical applications, with attention to optimal concentrations and operating conditions. These polymers have long served as the backbone of fracturing fluid systems due to their viscosity, availability, and ease of use. However, their performance is strongly influenced by reservoir conditions and formation chemistry, often resulting in degradation or adsorption within fractures.

### 2.3. Guar Gum

**Figure 1:**
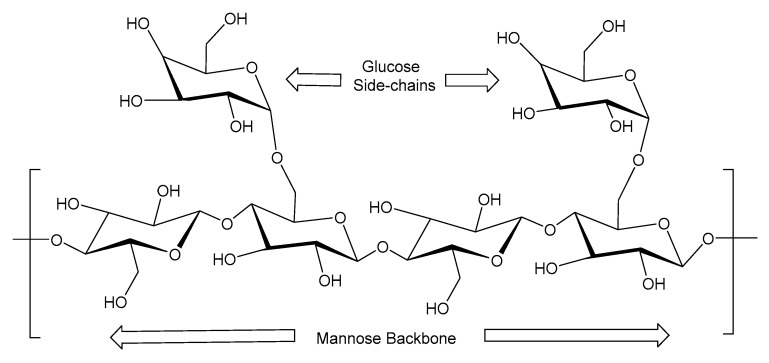
Guar gum structure, Mannose, and Galactose sides [56].

Guar gum is a neutral polysaccharide derived from the seeds of the guar plant (*Cyamopsis tetragonoloba*). It is obtained by drying and grinding the seeds to extract the endosperm, which contains galactomannan chains composed of a mannose backbone with galactose side groups in a ratio of approximately 1.6–1.8:1 [52,53], shown in Figure 1. This is rich in hydroxyl groups, and this molecular structure allows guar gum to form hydrogen bonds with water molecules, resulting in a stable, highly viscous gel. In its natural state, guar gum is neutral. The guar has been used extensively in fracturing operations due to its low cost, ease of preparation, and accumulated experience. However, lab experiments and field studies indicated that the guar gum has many drawbacks, including residue leftovers, which reduced the conductivity. The insoluble residue from processed guar is 10–14%, depending on the isolation method, which can block flow and reduce fracture conductivity. Chemically modifying guar gum can improve its performance [54]. The mannose-to-galactose ratio and the type of structural linkages influence the behavior of guar gum in fracturing fluids. Factors like temperature and breaker composition affect the rate and extent of this degradation and guar solubility in water. The locust gum residue had a mannose-to-galactose ratio of 5.2 and might have been soluble at 104 °C. However, the guar residue with a mannose-to-galactose ratio of 7.0 might not have been soluble until above 135 °C. The lower mannose-to-galactose ratio leads to high solubility, while a higher mannose-to-galactose ratio leads to low solubility and more residue. Guar gum is used in hydraulic fracturing because it forms highly viscous solutions at low concentrations and enhances fluid loss control. The guar is applied with 20–40 pounds per 1000 gallons of water, providing enough viscosity for proppant transport in shallow to moderate-depth wells with minimal temperature and pressure challenges. Cross-linking guar gum with covalent and hydrogen bonds increases viscosity and network stability up to 2000 mPa⋅s [55].

Ihejirika et al. (2015) [57] optimized the performance of guar gum in fracturing fluid against shear degradation, high temperatures, and residue formation. Results showed that higher guar concentrations and proper cross-linking improve fluid re-healing after shear, which is crucial for deep wells and high-stress operations. Guar fluids proved compatible with KCl brine but degraded in CaCl_2_; however, it has to be optimized to ensure effective proppant transport. The recommended hydration practice is to hydrate under moderate agitation for 10 min. Then, the crosslink borate solution should be added with acid and soda solution [57]. Guar viscosity drops quickly when breakers are added, but the polymer continues to degrade and can leave residue that slows cleanup. Modeling shows this degradation persists well beyond the initial viscosity loss, reducing overall recovery. Most breakers hydrolyze acetal linkages: breaking β-1,4 bonds on the backbone lowers molecular weight and viscosity, while breaking α-1,6 side-chain bonds raises the mannose-to-galactose ratio and reduces solubility. Consequently, breaking continues after the visible viscosity drop and can generate additional residue. A practical alternative is to use low-molecular-weight guar without an internal breaker, keep the fluid stable during pumping, and rely on borate crosslink pH reversibility and formation buffering to decrosslink and clean up, improving proppant-pack conductivity with less residue [58].

Guar gum is preferred among many polymers in hydraulic fracturing, due to its low cost, ease of use, and accepted viscosity values. It can work in high-salinity and high-temperature settings when properly formulated. However, key limitations include shear and microbial degradation, residue formation, and reduced conductivity in divalent-ion-rich environments [5,57,59]. Recent developments in polymer chemistry and cross-linking methods help overcome these limitations, making guar gum derivatives and synthetic polymers practical alternatives for use in fracturing operations. Many modifications and additives have been introduced to enhance the performance of guar and fracturing fluids, including the development of guar derivatives through oxidation in both basic and acidic environments, as discussed in the following sections.

### 2.4. Hydroxypropyl Guar

**Figure 2:**
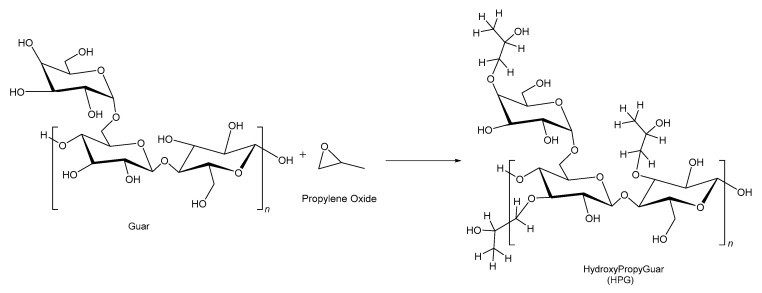
HPG preparation from Guar [61].

Hydroxypropyl guar (HPG) is a derivative of guar gum, a long-chain, high-molecular-weight polymer composed of mannose and galactose sugars. Figure 2 illustrates the natural guar and the process of change to HPG. The preparation of HPG involves processing guar beans to remove insoluble residues. Then, derivatizing the guar with propylene oxide in the presence of 4% sodium hydroxide as a catalyst, using either a slurry method with 75% aqueous isopropanol at 50–70 °C for 1–1.5 h, or a semi-dry method with minimal water. The mixture is then neutralized with acetic acid, washed with 75% aqueous isopropanol, and air-dried [60]. In the HPG production process, some hydroxyl groups are replaced with hydroxypropyl groups. This modification enhances its water and alcohol solubility and improves its thermal stability, although it remains neutral in its natural state. Modification to HPG reduces crosslinking sites, improving high-temperature stability and water solubility. Processing and washing cut water-insoluble residue to 2–4%. This modification also reduces the insoluble residues, minimizing damage at the formation face and in the proppant pack [17].

HPG works well with various additives that improve its performance. For example, cross-linking agents like borate significantly increase the viscosity of HPG solutions, making them more effective in transporting proppants during hydraulic fracturing. Borate-cross-linked HPG stays viscous and elastic between pH 7 and 12.5, but starts to break above pH 13. At very high pH, the polymer chains break down, reducing viscosity. Ion precipitation from sodium and borate further disrupts cross-linking, making the gel less stable [62]. Higher temperatures reduce viscosity by affecting hydration and loosening the polymer structure. Cross-linking counters this thinning effect, keeping the fluid thick and effective for hydraulic fracturing. With the right cross-linking additives, HPG can stay stable in wells up to 150 °C [63]. Both metal (i.e., zirconium) and salt (i.e., borate) are used with HPG as delayed and instant cross-linkers separately and together in the same system. Borate cross-linkers, such as sodium tetraborate (Na_2_B_4_O_7_), function effectively in alkaline pH (slightly below 9 to 11) and moderate temperature environments but lose efficiency in high-salinity or low-pH conditions due to reduced borate ion availability. Fan et al. (2017) showed that a system using 0.3 wt. % HPG and 0.8 wt. % Na_2_B_4_O_7_ achieved optimal viscosity and thermal resistance, with cross-linking time decreasing as cross-linker and polymer concentrations increased [64].

In a study by Al-Mohammed et al. (2007) [65] the degradation of high-pH borate fracturing gels made from guar gum and hydroxypropyl guar (HPG) was evaluated using sodium bromate and chlorous acid breakers under high-temperature, high-pressure conditions. HPG gels degraded much faster than guar gels and produced significantly less residue, which improved fracture clean up and more efficient well productivity. In contrast, guar gels left more insoluble residue, increasing the risk of blockage within the fracture and impairing conductivity, even at lower polymer loadings. The study also found that the surface tension of gel filtrates was independent of breaker type but consistently decreased with rising temperature [65]. In another study, other polymers, such as polyacrylamide-acrylic acid (P(AM-AA)) copolymers are incorporated with HPG to improve their properties in high salinity and high-temperature environments. These copolymers enhance hydrogen bonding with HPG, which limits molecular coiling and boosts water retention, leading to increased viscosity and viscoelasticity at temperatures above 120 °C. The presence of citric acid further supports fluid stability by counteracting the negative impact of ions on HPG hydration [66]. HPG performs better than natural guar gum in several ways. It offers improved thermal stability, produces less residue, and works well with cross-linking agents. Compared to guar, HPG maintains its viscosity more effectively under high temperatures and alkaline conditions, making it more suitable for challenging reservoir environments. It also breaks down more easily with breakers and can be further enhanced with additives to handle high salinity and temperature. However, its slightly higher cost is due to the additional processing steps involved in its preparation.

### 2.5. Carboxymethyl Hydroxypropyl Guar

**Figure 3:**
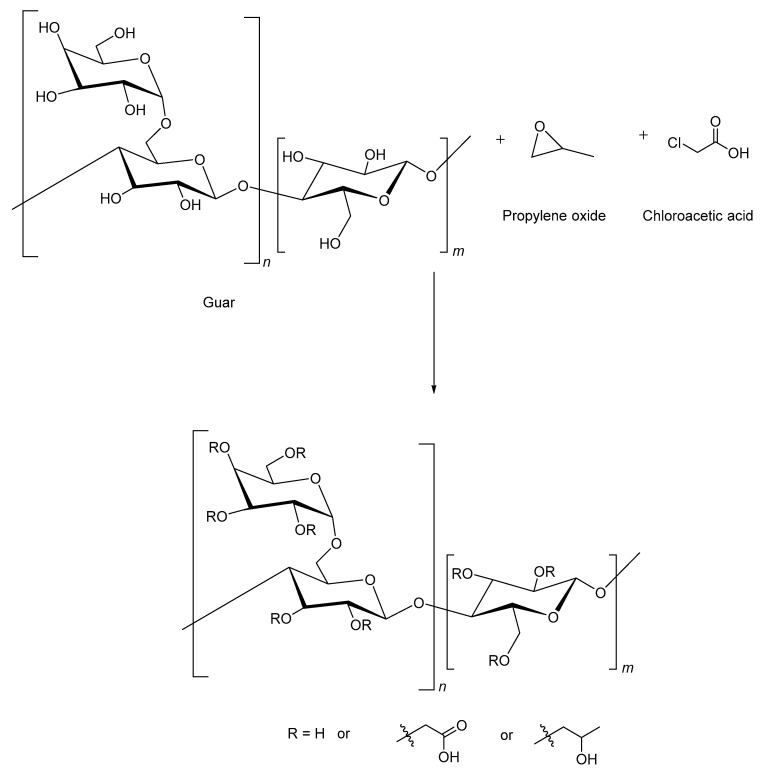
CMHPG preparation from Guar [69].

Carboxymethyl hydroxypropyl guar (CMHPG) is a modified form of guar gum with improved stability and minimal residue buildup. In this modification, carboxymethyl groups are introduced into the guar structure [67]. The added groups give the polymer a negative charge and make it anionic. The anionic charge of CMHPG depends on the level of carboxymethylation during processing [60]. The guar is treated with propylene oxide and monochloroacetic acid in sodium hydroxide. Controlling the concentration of sodium hydroxide is crucial for the proper modification of the introduced functional groups. Figure 3 shows the natural guar and the process of change to CMHPG [67]. There are two methods to prepare CMHPG: a slurry method with 75% isopropanol at 50–70 °C for 1–1.5 h and a semi-dry method with minimal water. The addition of chloroacetic acid determines the preparation of CMHPG instead of HPG. CMHPG leaves little residue, about 1%, making it cleaner than HPG and guar gum [60]. It is reported that CMHPG derivative is preferred in hydraulic fracturing treatments due to its efficient hydration and slower degradation when compared to other polymers, and has a wide range of pH tolerance [27,68]. Synthetic polymers are added to increase the intermolecular hydrogen bonds of CMHPG. Sodium thiosulfate scavenges oxygen, preventing oxidation of the polymer backbone, although it can also collapse the polymer conformation under certain conditions. CMHPG solutions exhibit shear-thickening behavior, reduce drag during pumping, and ensure efficient proppant transport. CMHPG forms strong, stable bonds when cross-linked with zirconium-based cross-linkers; these cross-linked polymers are suitable for high-temperature applications. Their performance deteriorates above 120 °C due to thermal and shear degradation; however, with the addition of synthetic polymers and high-temperature stabilizers like sodium thiosulfate, the thermal stability can be improved up to 177 °C [69].

Zirconate cross-linkers, often used in a delayed form, activate at elevated temperatures (>80 °C) and provide superior thermal stability and brine tolerance, though they require controlled pH and ligand balance to prevent premature gelation [70]. Different cross-linkers behaved differently in the study by Othman et al. (2024) [71]; they inspected the effect of zirconium delayed cross-linkers, borate instant cross-linkers, and dual zirconium-borate cross-linkers at 70 °C temperature and high salinity environments. GLDA chelating agents were added first to capture hardness cations, then the polymer was hydrated, followed by careful crosslinker addition. The same study highlighted that the dual cross-linkers performed better than the others [71]. CMHPG hydrates well at 5–6 pH and can cross-link at low and high pH levels. A study using NaBr-based high-density fluids found that CMHPG with optimized zirconium and borate cross-linker concentrations (0.7 ppm and 1.5 ppt, respectively) maintained viscosity and stability up to 177 °C, especially in alkaline conditions (pH 10–12). This range ensures better viscosity and stability of the fracturing fluid [72]. CMHPG performs better than both guar and HPG in hydraulic fracturing. It offers improved thermal stability, produces less residue, and hydrates well across a wider pH range. These features make CMHPG a preferred choice for challenging reservoir conditions.

### 2.6. Carboxymethyl Cellulose

**Figure 4:**
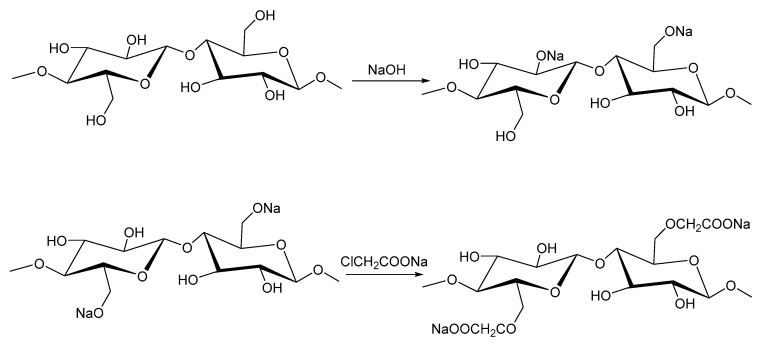
Synthesis of CMC [76].

Cellulose-based fracturing fluids come from modified plant cellulose. Cellulose-based fracturing fluids can be based on carboxymethyl cellulose (CMC) or hydroxypropyl cellulose (HPC), and are compatible with acidic and water-based systems. A low-molecular-weight cellulose derivative can be designed to give better solubility and faster dissolution through processes such as alkalization and etherification. A chemically modified cellulose polymer has improved thickening properties, greater temperature resistance, and minimal residue after gel breaking. Introducing carboxyl groups weakens the strong hydrogen bonds in cellulose, improving the partial separation of hydroxyl charges [73]. The β-1,4-glucan backbone is modified into water-soluble CMC through a two-step process: first, cellulose is treated with sodium hydroxide to form an alkali-cellulose complex; second, this complex undergoes etherification with sodium monochloroacetate, producing CMC, sodium chloride, and sodium glycolate. Etherification reactions improve their hydrophilicity and therefore solubility [74,75]. Figure 4 shows a simple representation of CMC preparation from cellulose. The modified cellulose can form a viscous gel when cross-linked at pH 5–7 [73].

Linear CMC viscosity ranges from 20 to 50 cP; however, with cross-linkers, it can reach 1000 cP. The cross-linked CMC systems are stable at temperatures up to 100 °C, but degrade at higher temperatures [75]. The CMC has low residues after the gel breaks down, which reduces formation damage and prevents secondary pollution. The CMC polymers naturally prevent the clay swelling; therefore, minimal stabilizer usage is needed. However, in tight formations, they can cause damage; therefore, careful preparation and concentration are recommended [73]. Chemical oxidizers are used to break the viscous CMC fracturing fluids. In a study by Scheffer et al., (2021) [77], a methanogenic enzyme system demonstrated effective degradation of CMC under oilfield-relevant conditions, including temperatures between 50 and 80 °C, salinities up to 20% (*w*/*v*), and pH 5–8. The extracellular enzyme fraction significantly reduced fluid viscosity under both oxic and anoxic conditions, indicating a strong potential for enzyme-based treatments to replace chemical oxidizers in removing CMC filter cakes during hydraulic fracturing operations [77]. CMC produces lower residue than guar and its derivatives and naturally helps prevent clay swelling, making it well-suited for cleaner fracturing operations. It performs effectively in water-based and acidic systems. However, it does not achieve the same viscosity levels as guar or CMHPG, has lower thermal stability than CMHPG, and requires careful handling in tight formations to minimize potential damage.

### 2.7. Xanthan Gum

**Figure 5:**
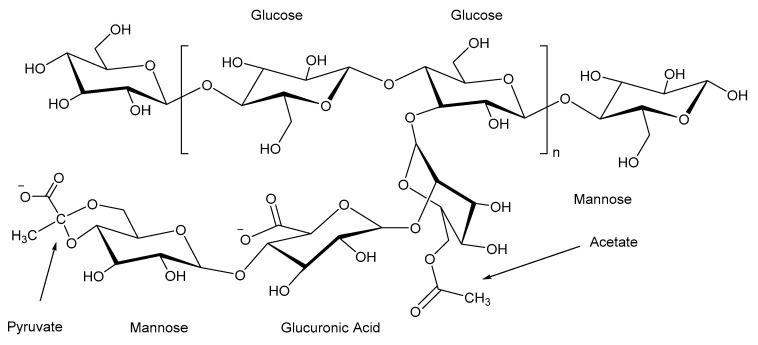
Xanthan gum structure [79].

Xanthan gum is a polysaccharide produced through the fermentation of Xanthomonas campestris, a process in which the bacteria metabolize simple sugars to create a β-1,4-linked glucose backbone. Trisaccharide side chains, consisting of mannose, glucuronic acid, and mannose, are attached to the backbone. These chains are modified with acetate and pyruvate groups, giving them a negative charge. These chemical modifications enhance the polymer’s solubility, stability, and ability to form highly viscous solutions. Therefore, the residue is considered minimal among the polymers used in hydraulic fracturing, with less than 1% residue. Xanthan gum hydrates effectively in cold and warm water through polymer particle swelling, dissolution, and interaction with water molecules. This hydration allows xanthan to achieve high viscosities, often exceeding 105 cP at low shear rates, even at concentrations as low as 0.05 wt. %. The polymer’s rigid double-helical structure, as shown in Figure 5, provides it with the mechanical strength necessary to form strong gel networks. Linear xanthan-based fluids typically have viscosities between 10 and 50 cP, while cross-linked systems, formed via ionic bonding with trivalent ions such as Fe^3+^ or Cr^3+^, achieve viscosities ranging from 500 to 5000 cP, offering enhanced elasticity and mechanical stability [78].

In hydraulic fracturing, xanthan gum is highly valued for its flexible chemical and mechanical properties. Its anionic nature allows it to interact electrostatically with metal ions, forming robust gel networks that maintain viscosity in saline or high-ion-content environments. Xanthan gum excels in drag reduction due to its shear-thinning behavior, which reduces frictional pressure during fluid pumping while maintaining sufficient viscosity to suspend and transport proppants. This ensures even distribution of proppants within fractures and prevents settling, even under high-pressure conditions. Xanthan also performs well in diverse water salinities and remains compatible with many additives, making it ideal for challenging reservoir environments. However, under high shear and elevated temperatures, xanthan begins to degrade, with viscosity loss occurring above 75 °C and thermal stability maintained up to 120 °C. When Xanthan is heated between 50 and 80 °C, its structure changes from an orderly helical shape to a more random, disordered coil. This transition makes the material more flexible, which can be beneficial. However, without stabilizers, this increased flexibility may reduce the material’s ability to perform well under very high temperatures. The strong carrying capacity and resistance to enzymatic degradation make xanthan gum a robust candidate for hydraulic fracturing treatments [78]. Xanthan gum also used as a gelling agent in foam-based fracturing fluids, caused significant permeability damage (up to 95%) in narrow natural fractures due to gel residue and filter cake buildup. Standard oxidizing breakers were largely ineffective against xanthan residues, requiring specialized breaker systems. Fluid leak-off was reduced with xanthan gels, though spurt loss increased when breakers were added without sand. Compared to polyacrylamide, xanthan posed a higher risk of formation damage under tested conditions [80]. Xanthan gum creates high viscosity even at low concentrations and performs well in salty water. It produces minimal residue, making it cleaner than guar and HPG and comparable to CMHPG. Xanthan also offers better shear-thinning behavior and flexibility compared to CMC. However, it is more sensitive to heat and can cause formation damage in narrow fractures due to filter cake buildup. It also needs specialized breakers for cleanup. Despite these drawbacks, xanthan is a reliable choice for challenging conditions involving high salinity and shear.

The polymers discussed, particularly guar derivatives, are widely used in hydraulic fracturing treatments. In 2012, the U.S. oil industry reportedly purchased approximately 300,000 metric tons of Indian guar gum, accounting for nearly 75% of India’s total production, with demand surging to the extent that prices doubled week by week due to panic buying [81]. Modified forms of guar, such as CMHPG, have become increasingly favored in North America, representing over 60% of polymer usage in newer fracturing fluid formulations due to their improved thermal stability and salinity tolerance [82]. In comparison, PAM consumption in the U.S. hydraulic fracturing sector is estimated at 75,000 metric tons annually, and a 2017 study found that 98% of 750 wells surveyed across six U.S. states used PAM, with each well consuming between 0.2 and 6 tons [83]. The following table provides a summary comparison of commonly used polymers, highlighting key aspects such as typical gel loading, preparation methods, thermal stability ranges, salinity tolerance, cost, and formation performance (Table 2).

### 2.8. Polymer Cost

The following Table 3 summarizes the costs of selected polymers commonly used in hydraulic fracturing. Most of the data are sourced from the [84]. Prices may vary depending on regional availability, grade, and application. This comparison highlights the cost differences among natural, modified natural, and synthetic polymers used in fracturing and related oilfield operations.

## 3. Other Fracturing Fluid Polymeric Additives

### 3.1. Friction Reducers

**Figure 6:**
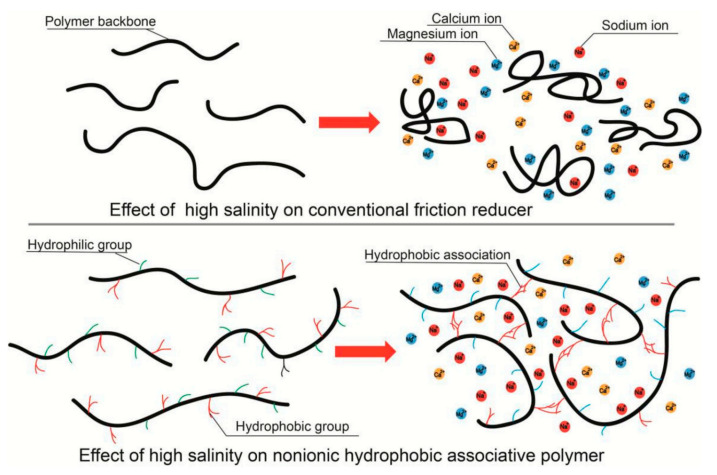
Conventional vs. nonionic hydrophobic polymers. Reprinted with permission from (Yang et al., 2019) copyright (2025) American Chemical Society [88].

Friction reducers (FRs) are used in hydraulic fracturing to minimize frictional pressure losses when high pumping rates are needed. They are complex, high-molecular-weight water-soluble polymers like sodium acrylate-acrylamide polymer (C_3_H_3_NaO_2_) and polyacrylamide (C_3_H_5_NO)_n_ [38]. During pumping, the polymer chains of FRs align with the flow to reduce turbulence and drag force. They are essential in slickwater fracturing fluids, these fluids are low-viscosity fluids pumped at high rates to create fractures in shale and tight formations. FRs are typically added with concentrations ranging 0.25 to 2 gal/1000 gal. The FRs come in anionic, cationic, and nonionic formulations, and are available as emulsions, dry powders, and biodegradable options. They are designed to perform in different water chemistries, including high-salinity environments. Effective FRs have high molecular weight, shear stability, temperature resistance, and salt tolerance, ensuring proper proppant transport and well-stimulation in challenging downhole conditions. However, they face degradation challenges with very high shear and are sensitive to very high salinity or temperature, and further improvements are required [86]. To address high salinity, salt-tolerant FRs have been developed using modified polyacrylamide formulations. These FRs maintain high efficiency in produced water with TDS levels up to 300,000 ppm. They resist polymer chain folding caused by divalent ions, ensuring stable drag reduction. Figure 6 shows the comparative effect of high salinity on conventional FRs versus nonionic hydrophobic associative polymers [87].

Bio-based FRs, cost-effectiveness, and smart polymers with better performance in unconventional oil and gas extraction are developed to solve some of the issues faced in harsh environments. FRs are commonly formulated as inverse emulsions, where the polymer is dispersed in an oil phase to enhance solubility, stability, and controlled release. The performance of friction reducers is affected by factors like molecular weight, charge type, and charge distribution, especially in high-salinity environments where ions like calcium and magnesium can interfere with polymer hydration and dispersion. It is also important for these additives to be compatible with other fracturing chemicals, such as biocides, scale inhibitors, and surfactants, to maintain fluid efficiency and stability. FR efficiency is typically measured using friction loop testing, with the best results coming from high Reynolds numbers (around 150,000), where higher flow rates help improve dispersion and reduce drag [86,89]. Temperature plays a significant role in FR performance, with some formulations remaining stable up to 40 °C, while others degrade at higher temperatures, requiring specialized surfactant packages for enhanced durability. Salt-tolerant FR formulations, particularly those designed for high-TDS environments, exhibit rapid hydration and consistent performance under challenging field conditions. Innovations in breaker surfactants, polymer charge engineering, and bio-based solutions continue to enhance FR efficiency, improving adaptability to diverse well environments while addressing environmental and operational concerns [90]. FR selection depends on well conditions, water chemistry, and operational requirements, with ongoing advancements aimed at improving their efficiency and environmental compatibility in hydraulic fracturing applications. There are many types of polymeric friction reducers used in hydraulic fracturing treatments.

Polyacrylic Acid (PAAc): (A) non-toxic, high-molecular-weight synthetic polymer of acrylic acid, available as a white solid or a 50% active dispersion in mineral oil. It disperses and solubilizes easily in water but is highly sensitive to divalent cations (Ca, Mg, Fe), leading to rapid precipitation in hard water. Other applications include adsorbents for disposable diapers, ion exchange resins, adhesives, and thickeners in pharmaceuticals, cosmetics, and paints. (B) Polyacrylamide (PAAm): Formed from acrylamide subunits, PAAm is non-toxic, though unpolymerized acrylamide is a neurotoxin. As a solid, it hydrates more slowly than PAAc but is less sensitive to divalent cations. It is typically delivered as a 50% active suspension emulsified in mineral oil. PAAm is difficult to break and can gel 15% HCl, potentially damaging the reservoir rock and proppant pack. However, optimized breaker treatments have been reported to improve its use in slickwater fracturing [61]. Other applications include wastewater treatment, papermaking, soil conditioning, and soft contact lenses. (C) Partially Hydrolyzed Polyacrylamide (PHPA): The most commonly used friction reducer, PHPA is produced by reacting sodium acrylate with acrylamide, hydrolyzing approximately 30% of the acrylamide groups. This improves water solubility and compatibility with cationic minerals. Marketed as a 50% active dispersion in mineral oil, PHPA is widely used as a flocculant in water treatment and papermaking, making it the least expensive and most prevalent FR in hydraulic fracturing. (D) Acrylamide Methyl Propane Sulfonate (AMPS): Designed for stability in hard water and high-temperature environments, AMPS resists precipitation by cationic mineral salts and remains functional across a broad pH range, making it suitable for energized fluids containing CO_2_. Its sulfonate group also enhances scale inhibition properties. Typically sold as a 50% active emulsion, AMPS is also used in electrocardiogram gels, concrete plasticizers, and water treatment coagulants [61].

### 3.2. Scale Inhibitors

Scale formation is a significant challenge in the oil and gas industry, leading to equipment failure, increased costs, formation damage, reduced permeability, and hence the production [91]. Scale inhibitors (SIs) are chemical additives used in hydraulic fracturing to prevent mineral scale formation. When using seawater in hydraulic fracturing, scale formation is primarily caused by the interaction of connate water, which contains calcium and barium ions, with sulfate-rich seawater. The most common scales in seawater-based fracturing fluids include calcium carbonate (CaCO_3_), which increases with temperature and pH changes, calcium sulfate (CaSO_4_, gypsum), barium sulfate (BaSO_4_, barite), and iron scales like ferrous carbonate (FeCO_3_) [92]. Scale inhibitors (SIs) attach to scaling ions, preventing crystal formation and deposits. They need to work well with other fracturing additives like surfactants and biocides and should stay in the formation or proppant pack for long-term release. Their effectiveness depends on flow rate and temperature, with sulfonate-based inhibitors performing best at high temperatures. Nanofiltration (NF) treatment with advanced scale inhibitors has successfully controlled barite scale in high-barium and sulfate-rich environments [93]. Including SIs in fracturing fluids helps them distribute evenly in fractures, offering lasting protection against scale and lowering expenses. Tracking SI levels in produced water allows for adjustments to improve treatment efficiency. Examples from field applications demonstrate that wells treated with SIs experience higher productivity and fewer shutdowns due to scale issues, proving them to be a practical and economical choice for sustained operations [94].

Managing the pH levels and using the scale inhibitors is necessary to prevent scale formation. Common scale inhibitors are created by combining monomers such as acrylic acid, maleic acid, phosphates, and phosphonates like HMDTMP. Gelled mixtures of phosphonate and seawater can stop the calcium scale at high temperatures while pH-adjusting agents help control the magnesium scale. Adding phosphonate or carboxylate groups improves the ability to bind metals, and cross-linking increases heat resistance. The polymers are then neutralized, mixed with stabilizers like ethylene glycol, and turned into liquid, emulsion, or solid forms. The final products are tested to make sure they work well under different salt levels and temperatures and effectively prevent scale [94].

### 3.3. Comparison of Cross-Linked System to Slick Water System

Slickwater is a low-viscosity fracturing fluid composed of water, friction reducers, and some fracturing fluids additives such as biocides, scale inhibitors, surfactants, clay stabilizers, and proppants. It reduces friction during high-rate pumping, allowing fluid injection at lower pressures and creating long, complex fractures. Friction reducers, usually polyacrylamides, come in anionic, cationic, or nonionic forms, with anionic types being the most common due to their compatibility with various water chemistries. Cationic friction reducers like quaternary ammonium polymers are used in high-salinity environments, while nonionic types work well for low-residue applications. Slickwater is widely used in shale formations and unconventional reservoirs, where it generates extensive fracture networks. Slickwater has a low viscosity of 3–5 cP, uses minimal friction reducer concentrations (0.025–0.05 wt. %), and is pumped at very high rates, often exceeding 60 bbl/min. Compared to cross-linked fluids, which have a much higher viscosity (500–5000 cP) and are pumped at lower rates (less than 40 bbl/min), slickwater forms long, complex fractures with minimal residue and lower costs. Cross-linked fluids, which rely on polymers like guar, HPG, CMHPG, or CMC at 20–40 lbs/1000 gallons, are better suited for deep, high-pressure, high-temperature reservoirs where vertical fracture propagation and substantial proppant transport are required. However, they come with higher costs and potential residue concerns. In high-salinity environments, surfactants can enhance friction reducers [95,96].

## 4. Polymers’ Interaction with Fracturing Fluid Components

Polymers in fracturing fluids exhibit several mechanisms, mainly hydration, cross-linking, and breaking. Many additives and conditions affect these mechanisms. For example, the pH affects the hydration and cross-linking of the polymer and can be controlled by adding buffers. The polymers cross-link with the addition of cross-linkers, and the gel breaks with the help of breakers. The salinity and temperature affect the cross-linking and breaking. This part of the review helps to understand how polymers function with different additives under different conditions.

### 4.1. Hydration

The first critical step in fracturing fluid preparation is polymer hydration, where water molecules penetrate and swell the polymer chains, increasing viscosity. Hydration efficiency depends on several factors: pH, temperature, polymer concentration, and water salinity. It is most effective at the lower pH, close to 5, while extreme pH levels can reduce the hydration efficiency due to polymer degradation or ion interference [97]. Acidic conditions (pH < 5) lower guar-derivative solubility by protonating anionic groups, collapsing the coils, shrinking the hydrodynamic radius, and even causing precipitation. Under mild alkalinity, deprotonation increases negative charge and coil expansion, so viscosity initially rises; at higher alkalinity (>10), side chain/amide hydrolysis and increased solution mineralization inhibit hydration and reduce the hydrodynamic radius, so viscosity then falls. The hydration behavior of polymers varies between freshwater and seawater. At pH 5–6, HPG reaches maximum hydration after 50 min in seawater versus 5 min in freshwater because high salt slows water penetration. Temperature also affects hydration, with higher temperatures (38–49 °C) resulting in lower viscosity, especially in saline waters. At 49 °C, viscosity is markedly lower than at 25 °C, requiring optimized hydration conditions [68,98]. Water-soluble polymers are essential for enhancing performance and thermal stability and fall into three categories: biopolymers, synthetic polymers with strong covalent bonds and functional side chains, and composite systems, including synergistic blends and nanocomposites. As oil and gas exploration moves into harsher environments, research focuses on enhancing fracturing fluid performance through molecular modifications, advanced cross-linkers, nanotechnology, and cost-effective synthetic polymers [99].

#### Hydration in Saline Waters

To promote sustainability and due to freshwater scarcity, the industry has developed freshwater-less fracturing fluids that utilize alternative sources such as seawater, produced water, and sewage water [29]. However, saline waters encounter challenges due to ions like calcium, carbonate, and sulfate, which can cause precipitation, scaling, and corrosion; they affect permeability, and reduce fluid viscosity, and stability [100]. The calcium and magnesium ions affect the efficiency of additives like pH buffers and breakers. These ions interact with the polymer chains, reducing their hydration efficiency. Several additives are introduced to smooth the water and reduce the electrostatic interactions between cations and the polymer, such as chelating agents. This allows the polymer chains to spread more effectively and hydrate faster. This method is useful for offshore fracturing operations by using seawater-based fracturing fluid [94]. The levels of these ions are used to determine the hardness of saline waters, which is measured as calcium carbonate equivalents, and affects the hydration and breaking mechanisms. A higher pH is needed in high temperatures to maintain the thermal stability of the fluids, as the gels are weak at such temperatures. However, at high pH levels, the associated ion can form scale deposits; therefore, scale inhibitors are necessary. The CMHPG is superior to HPG in high-salinity environments, as it hydrates within 10 min. Higher polymer concentrations increase viscosity, but saline water reduces maximum viscosity compared to freshwater [39,68,101,102,103,104]. Magnesium hydroxide starts to precipitate at pH 10.4 at standard conditions, but as temperature rises, it precipitates at a lower pH. CMC has strong thermal stability with saline water, while Xanthan performs very well in high salinity conditions due to its anionic nature and robust gel structure. The performance of these polymers can be enhanced with suitable additives. Synthetic polymers offer alternatives with specific advantages, such as minimal residue and self-breaking properties. However, they remain sensitive to high-valence ions, therefore careful pH management and fluid compatibility should be considered [105].

### 4.2. Polymers Cross-Linking

A well-designed fracturing fluid must achieve the right viscosity at the appropriate stage. If the fluid becomes too viscous prematurely, it can lead to high pumping pressures or structural breakdown due to intense shear forces in turbulent flow. If the fluid has low viscosity, huge energy is required to break the formation, and it will not be able to carry the proppant. To achieve the required viscosity, increasing polymer concentration is an option; however, this approach is inefficient as it raises costs and pumping pressures and increases the residues, which damage the induced fractures [20,26,29]. A more effective solution is to use the cross-linkers, which chemically bond with the polymers to enhance viscosity and elasticity without increasing polymer content. This transformation converts the fluid from a simple viscous state to a viscoelastic one, improving proppant suspension and transport while maintaining structural integrity. Cross-linking forms a three-dimensional gel network that strengthens the fluid. The cross-linking mechanisms are either chemical or physical cross-linking [47]. Both mechanisms are encountered in fracturing fluid preparations. In hydraulic fracturing operations, cross-linkers are generally categorized into salt-based and metal-based systems, each offering distinct activation mechanisms and performance characteristics. The performance of borate and zirconium crosslinkers in fracturing fluids varies significantly with temperature, pH, and polymer type.

#### 4.2.1. Salt Cross-Linkers

**Figure 7:**
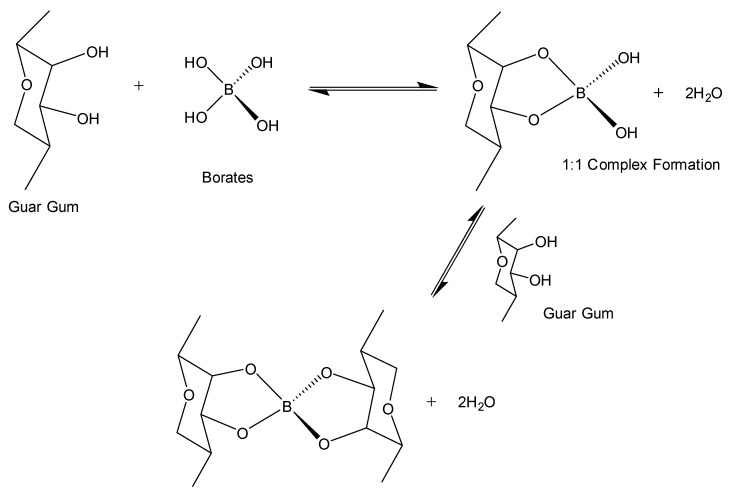
Equilibrium of borate ion on a Guar Gum to form a cross-linked gel [110].

**Figure 8:**
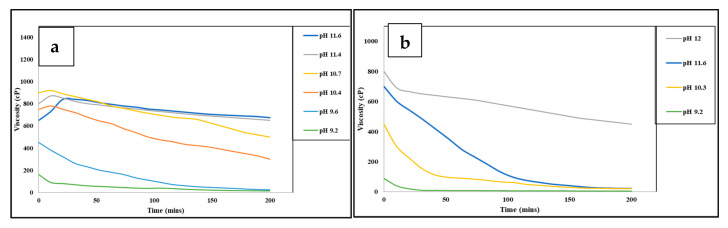
40 lbm/1000 gal guar with different borate cross-linker concentration (different pH) at shear rate 80 s^−1^ and temperature (**a**) 121 °C (**b**) 135 °C [109].

The borate cross-linkers are initially insoluble minerals like borax, which dissolve in water with heat or mixing. This releases borate ions gradually, ensuring cross-linking happens at the right time, to prevent early thickening. Borate ions bond with hydroxyl groups in guar-based polymers, forming reversible bonds. In HPG, they bond with cis-diol groups, creating a flexible gel. In CMHPG, carboxymethyl groups improve ionic interactions, boosting cross-linking and thermal stability, especially in saline environments. Borate salt cross-links guar when borate ions react with cis-diol groups in guar’s backbone, forming reversible bonds that create a three-dimensional gel network. Figure 7 shows borate ion cross-links guar gum in a 1:1 complex. There are two types of complexes: a 1:1 complex, where one borate ion binds to one cis-diol group, forming a weak initial cross-link, and a 2:1 complex, where one borate ion bridges two cis-diol groups, either within the same polymer chain or between different chains, resulting in a stronger gel network. These cross-linking mechanisms enhance elasticity, shear tolerance, and gel strength. Borate-based systems are cost-effective, offer good cleanup, and exhibit high shear tolerance with re-healing properties [106,107]. However, their high pH may cause downhole issues such as clay swelling and scale formation, making pH buffering within the range of 8–10 essential for stability. Below this range, borate remains inactive, while above pH 10 and 11 in saline conditions, the gel structure may destabilize. At standard conditions, borate cross-linkers are instant cross-linkers, while at elevated temperatures, they function as delayed cross-linkers because their ion release depends on temperature. Maintaining performance above 94 °C often requires pH adjustments or stabilizing agents. Borate cross-linkers help control gel formation by managing solubility [24,104,108]. According to Harris (1993) [109], borate cross-linking (shown in Figure 8) relies heavily on pH and temperature, where higher pH values (above 9) and high temperatures between 121 °C and 135 °C support strong gel structures and viscosity retention. However, as temperature increases, higher borate concentrations are required to compensate for reduced crosslink density and maintain fluid stability [109].

#### 4.2.2. Metal Cross-Linkers

Metal cross-linkers, such as zirconium, titanium, and aluminum, form covalent bonds with carboxyl groups in CMHPG, creating strong gel networks suitable for high-temperature and high-salinity conditions. The carboxymethyl groups in CMHPG enhance interactions with zirconium ions, resulting in a stable and elastic gel structure. These cross-linkers function effectively across a broad pH range (2–12) and temperatures below 38 °C to over 204 °C, maintaining viscosity in acidic and saline environments [26,108]. Zirconium oxychloride, acetic acid, and triethanolamine are often used to create a zirconium cross-linked system. These systems can exhibit delayed activation, as their effectiveness depends on specific fluid conditions such as temperature, pH, and salinity. The polymer type and conditions affect cross-linker performance; the same metal cross-linker can act instantly with some polymers but show delayed activation with others. Delayed activation happens through chemical binding. Metal ions attach to chelating agents or ligands, staying inactive until the fluid reaches a certain pH or temperature. Then, the bonds break, releasing the metal ion to cross-link with the polymer. Buffers like sodium carbonate, sodium hydroxide, or acetic acid adjust pH to optimize cross-linking [24,69].

**Figure 9:**
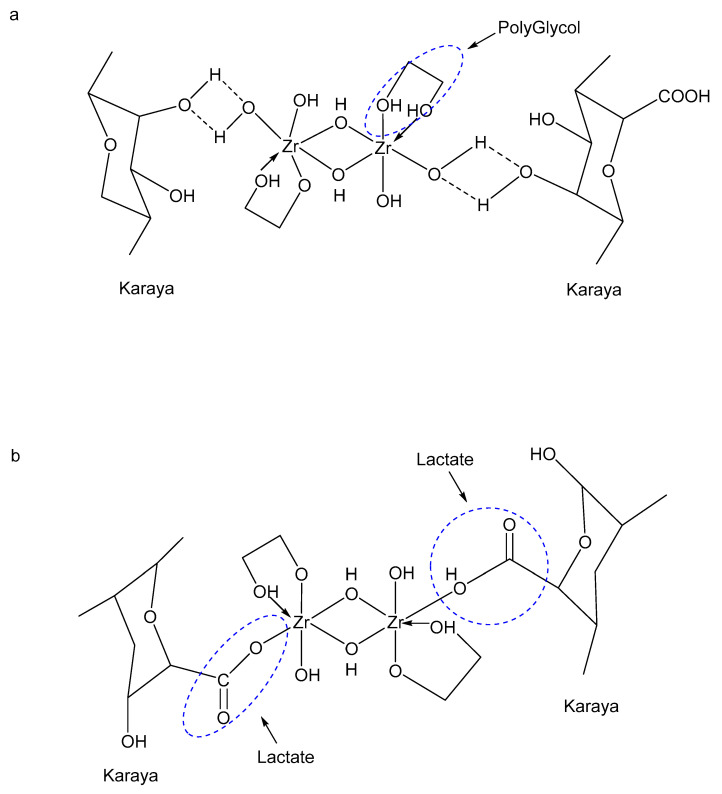
Zr-cross-links Guar with (**a**) Lactate, (**b**) propylene glycol [113].

Figure 9 shows zirconium cross-links with the Karaya end of guar using ligands like lactate, propylene glycol, and triethanolamine. Hydrogen bonds form with propylene glycol, while covalent bonds form with lactate, showing different bonds with different ligands [111]. A simple test to determine whether instant cross-linking has occurred involves assessing the gel’s ability to retain its form under normal conditions, indicating rapid viscosity development. Choosing the right metal cross-linker depends on reservoir conditions, such as temperature, salinity, and pH, to ensure optimal gel performance and stability during fracturing. Almubarak et al. (2020) [112] found that zirconium crosslinkers with lactate and propylene glycol (Zr-La-PG) work best with CMHPG fluids at 93–149 °C, giving good thermal and shear stability. However, this same crosslinker does not perform well with synthetic polymers, where another type, Zr-TEA-La, works better. The results also show that the type and order of ligands in the crosslinker affect how well the fluid thickens and how fast it crosslinks [112]. This means it is important to choose the right crosslinker based on the type of polymer and the conditions of temperature and salinity for the best results.

### 4.3. Polymer Breaking

Breaking cross-linked gels after placing the proppant allows efficient fluid flowback and minimizes formation damage. Breakers are materials that absorb cross-linkers, leaving minimal residual cross-linkers in the fluid. Breaking the fluid at the correct time improves the fracture conductivity; however, improper breaking can affect proppant placement and lead to premature screen-out. Therefore, the selection breaker and its timing are critical. Breakers are categorized into oxidative, enzymatic, delayed, and hybrid systems, that is, both oxidative and enzymatic, each with distinct chemistry and activation mechanisms. The breakers can be introduced to the fracturing fluid in two ways: internal breaker addition, in which they are mixed directly into the fluid during surface preparation. They initiate gel breakdown without contacting the reservoir fluids, ensuring predictable viscosity reduction. This method is commonly used in viscoelastic fluids, preventing viscous emulsions and facilitating cleanup [32]. The second way is external breaker addition, the breakers are introduced during flowback to help break down any remaining gel structures. External breakers are especially beneficial in high-temperature environments where maintaining fluid stability is critical before reducing viscosity [36]. The following paragraph discusses the types of breakers used with fracturing fluid treatments.

#### 4.3.1. Oxidative Breakers

Function by cutting the polymer backbone through oxidation reactions. Common oxidizing agents include ammonium persulfate (NH_4_)_2_S_2_O_8_, and sodium bromate NaBrO_3_, both of which generate free radicals that break down polymer chains. These breakers are widely used in both borate and metal cross-linked systems. Their activity is temperature-dependent, with higher temperatures accelerating polymer degradation. In borate cross-linked systems, oxidative breakers disrupt the reversible di-diol bonds between borate ions and hydroxyl groups on polymers like HPG and CMHPG. In metal cross-linked fluids, such as zirconate cross-linked CMHPG, oxidative breakers sever covalent bonds between metal ions and carboxyl groups, a process often enhanced by pH buffers like acetic acid [28,108]. These breakers were tested at temperatures of approximately 60 °C and 93 °C and demonstrated temperature-dependent degradation behavior. The gel systems were adjusted to a pH of approximately 5–6 for hydration and 10–11 for crosslinking, indicating the breakers function within that pH range. Breaker concentrations varied and were tested up to 10 gal/Mgal, with results showing that higher concentrations accelerated the degradation of the guar polymer gel [114].

#### 4.3.2. Enzymatic Breakers

**Figure 10:**
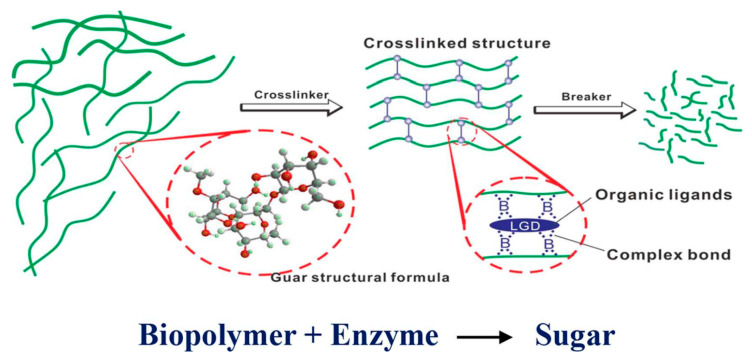
Enzyme breaking mechanisms for biopolymers. Reprinted with permission from (Ghosh et al., 2021) copyright (2025) American Chemical Society [87].

Enzymatic breakers, such as cellulase, function by hydrolyzing the polymer backbone, specifically targeting natural polymers like guar derivatives. Unlike oxidative breakers, enzymatic breakers operate under milder conditions, making them environmentally friendly and less aggressive. They are polymer-specific and self-regenerating, ensuring gradual viscosity reduction while maintaining equipment integrity. Enzymes are particularly effective in borate cross-linked fluids because they degrade guar-based gels without affecting other components [28,36]. They operate at temperatures 40–70 °C and a pH of 7–8.5. These breakers are not suitable for harsh conditions. Figure 10 shows how an enzyme breaks the biopolymer macromolecules.

#### 4.3.3. Delayed Breakers

Delayed breakers are formulated to activate at a controlled rate, ensuring viscosity reduction occurs only after proppant placement. These include surface-modified nanoparticles and encapsulated oxidizers that slowly release active agents. This controlled release prevents premature viscosity loss and ensures efficient cleanup. Conventional breakers can cause rapid viscosity loss with saline systems, leading to poor proppant placement. Nanoparticles provide a more controlled viscosity reduction, balancing fluid stability with effective cleanup [33,34]. Laboratory measurements alone cannot predict breaker performance in real fracturing conditions. Recent studies show that reservoir pressure can reduce viscosity by up to 80% compared to lab values. Fuller (2017) [115] suggested a method that uses pressure to change how borate cross-linked gels behave with temperature. This causes the gels to lose their thickness permanently [116]. These systems operate at moderate to high temperatures up to 160 °C [117]. A precise delay can be achieved with oxidizer loaded into mesoporous silica nanoparticles and sealed with a paraffin shell that melts at the target temperature. At 85 °C, paraffin-coated carriers released 0.00% at 0.5 h, versus 25.86% for uncoated; by selecting the wax melting point and nanoparticle pore size/coating thickness, the release profile can be tuned to the operation [118].

## 5. Formation Damage and Environmental Issues

### 5.1. Formation Damage from Polymers

Guar gum and its derivatives, including HPG and CMHPG, often leave residues that damage the induced fracture and proppant pack [119]. The guar gum has more residues than the other guar derivatives; after it breaks, it forms insoluble mannose helix structures and contains insoluble ash. The crushed proppants, due to mechanical stress, react to formation water and fracturing fluid, and precipitates occur between the proppants and formation fluids. The crushed minerals dissolve and re-precipitate, and reduce the permeability and porosity of the proppant pack [120]. Simultaneously, stress-induced proppant embedment into the fracture face leads to compaction and pore blockage caused by the migration of fine particles [121]. These residues can affect the wettability of proppant surfaces and nearby rock, which negatively impacts the hydrocarbon flow in formation [122]. Over time, the combined effects of proppant compaction and chemical breakdown can reduce permeability by as much as 75% under reservoir conditions [123]. Altogether, these mechanical and chemical interactions lead to severe formation damage and changes in fluid flow behavior.

**Figure 11:**
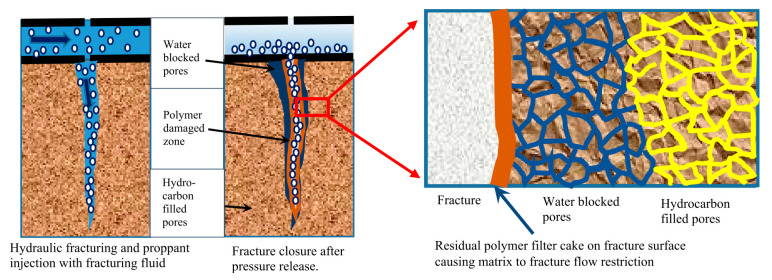
Fracture permeability damage caused by residual polymers and water blockage. Reprinted with permission from (Ghosh et al., 2021) copyright (2025) American Chemical Society [87].

HPG, while producing less residue, can still contribute to minor blockages if gel breaking is incomplete. CMHPG, due to its enhanced hydration and breaking properties, leaves minimal residue. All these polymers form filter cakes that reduce fluid loss but may lessen the flowback if not effectively removed. Incomplete gel breaking is a common challenge, especially in low-temperature or high-salinity environments, necessitating oxidative breakers like ammonium persulfate or enzyme-based systems tailored for each polymer. These polymers can intensify clay swelling and fines migration in water-sensitive formations unless mitigated with stabilizers like potassium chloride. Guar-based fluids also risk emulsions and hydrocarbon phase trapping due to incomplete cleanup. While guar and HPG can exhibit microbial degradation, CMHPG is more resistant to bacterial growth. Mitigation strategies to reduce damage include low-residue derivatives, optimized breakers, and biocides to address biodegradation and cake formation. Additionally, advanced fluid formulations further enhance thermal and saline resistance [124,125,126,127]. Guar gum and its derivatives adsorb onto quartz surfaces in sandstone reservoirs mainly through hydrogen bonding, reducing the permeability and damaging the formation. Adsorption increases with divalent salts and low pH. Yin et al. (2018) [128] studied guar gum adsorption from hydraulic fracturing fluids onto reservoir rocks. They inspected the methods that minimize the adsorption. The adsorption was quantified using a spectrophotometry method, which showed that high pH, elevated temperatures, monovalent salts, ethane diol, and HPAM additives effectively minimized adsorption [128]. Figure 11 shows the permeability damage caused by polymer residues, causing filter cake and water blockage.

Cellulose-based polymers, such as CMC and HEC, are known for their low residues and efficient gel-breaking capabilities. Cellulose derivatives are suitable for multiphase reservoirs, they do not form emulsions or trap phases in hydrocarbon-rich environments. Although these derivatives have less microbial activity, biocides are necessary in prolonged operations. The filter cakes created by these polymers support fracture integrity and minimize flowback issues. These polymers are also compatible with clay stabilizers used with clay-rich formations, which prevent swelling and fine migration. However, these filter cakes sometimes cause cleanup issues;; therefore, other additives are needed. Mitigation strategies include the use of enzymatic breakers and pH control to enhance fluid cleanup and performance, even in high-salinity conditions [124,125,126,127]. Compared to guar derivatives, Xanthan gum leaves a minimal residue, reducing the risk of pore blockage. It forms thin, elastic filter cakes that improve the fracture integrity and reduce fluid loss. Gel breaking is typically efficient with oxidative breakers, though high-salinity environments may present additional challenges in fluid rheology. Xanthan gum is highly resistant to interaction with clay and fines. Gel retention in low-temperature reservoirs causes severe permeability loss. It rarely forms emulsions, making it ideal for saline or hydrocarbon-rich reservoirs. However, xanthan is biodegradable, and the use of biocides is a must as microbial degradation can lead to prolonged formation damage. Mitigation strategies for xanthan include tailored breaker formulations and careful polymer concentration design to ensure efficient cleanup and minimal environmental impact [124,125,126,127].

### 5.2. Environmental Effects

The environmental impact of polymer usage in fracturing fluids and their residue is a serious concern. Synthetic polymers are riskier than natural polymers because they can break down into harmful substances like acrylamide, which may contaminate water for a long time and affect the food chain [129,130]. If these polymers spill into the ground, they may degrade into microplastics, making the soil hydrophobic and reducing water infiltration, which negatively affects plant growth [131,132]. If the produced water containing the polymer’s residues is reused for irrigation, it can harm soil structure and lower crop yields. Mixing polymer residues with other fracturing chemicals like biocides and surfactants can also create complex compounds that are hard to break down naturally [129,130]. Spills in seawater threaten aquatic life due to the toxicity of these polymers [133]. To address these, developing eco-friendly polymers that leave less residue and improving wastewater treatment can be part of the solutions. Enforcing strict regulations and safety measures, besides applying closed-loop recycling systems for fracturing fluids, can aid in reducing spills and environmental damage [134]. Public awareness campaigns can help in this regard. Enzyme-based breakers are emerging as a greener alternative to chemical oxidizers because they break down polymers efficiently without creating harmful waste, and they are safer for both equipment and rock formations [28,135].

## 6. Recommendations and Prospects

Generally, the polymeric fracturing fluid should be designed to enable precision-timed cleanup with temperature-activated breakers, nanoparticle carriers; therefore, the viscosity is preserved during placement and then drops rapidly on cue [118]. Also, in this fluid, the non-freshwater bases (saline waters) should be prioritized, with compatibility materials such as chelating agents and scale-inhibitors [43]. Likewise, pair the fluids with intelligent, real-time blending/monitoring to control the fluid properties. Finally, incorporate the nanoparticle-enhanced VES and seawater-tolerant clean-fluid development for high-T/High-TDS service [136], with field pilots to validate performance, residue, and economics [137]. Besides the materials added to tackle the temperature and salinity, early-stage pH control enhances polymer hydration, and raises the viscosity [118].

Selecting the right polymers for hydraulic fracturing is crucial for fluid performance, proppant transport, and formation stability. Guar gum derivatives, CMC, and xanthan gum provide viscosity and shear resistance but may leave residues that reduce permeability. Synthetic polymers like PAM offer cheaper alternatives but raise environmental concerns due to degradation byproducts like acrylamide. Balancing viscosity, cost, and formation impact requires optimizing polymer concentration and cross-linking efficiency. Temperature and salinity affect polymer performance, making salt-tolerant and thermally stable options like CMHPG and modified polyacrylamides necessary. Nanocomposites and associative systems can improve fluid stability while reducing environmental risks. Efficient breakers help minimize polymer residue and maintain fracture conductivity. Research continues on enzyme-based breakers and biodegradable viscosifiers to enhance sustainability. As produced water and seawater become more common in fracturing fluids, polymers must be designed to perform well in high-salinity conditions [138].

Improving the efficiency and sustainability of hydraulic fracturing requires a dual focus on eco-friendly practices and innovative fluid design. One key step is replacing high-residue polymers with biodegradable or low-residue synthetic alternatives, which maintain performance while lowering environmental harm. Advanced solutions like nanoparticles, viscoelastic surfactants (VES), and foam-based fluids can enhance proppant transport, cut water usage, and prevent formation damage. When working with alternative water sources, such as produced water or seawater, it is essential to choose polymers and cross-linkers that perform well in high-salinity conditions to ensure stability in the reservoir. Before field application, thorough compatibility testing helps prevent operational issues. Real-time monitoring and reservoir analysis, paired with adaptive polymer selection, fine-tune viscosity, thermal stability, and proppant placement. Beyond that, advancing biodegradable polymer technology, improving post-fracturing cleanup, and adopting closed-loop recycling systems can further minimize environmental risks and promote responsible resource extraction [25,83,139].

Guar gum and its derivatives remain the industry standard for building and breaking viscosity in hydraulic fracturing—a tried, tested, and reliable system. Despite certain drawbacks, guar-based fluids continue to dominate due to their long-standing performance and extensive field validation. While newer polymers have emerged, few have matched the consistent success and trust earned by guar and xanthan derivatives over decades of use. In contrast, synthetic and alternative biopolymers face notable challenges, including higher costs, limited availability, and environmental concerns. Some also struggle under extreme downhole conditions, which makes their widespread adoption more difficult [140]. Synthetic polymers such as PAM, HAPAM, and PAM-PEI are showing strong potential as alternatives to guar in hydraulic fracturing treatments. However, they are currently used primarily as additives. PAM is valued for its fast hydration, thermal stability, and moderate salt tolerance. It delivers sufficient viscosity for proppant transport while minimizing residue, which helps reduce formation damage and preserve fracture conductivity. It is also cost-effective, priced at approximately $2/kg compared to $5 for HPG and $10 for CMHPG. HPAM performs well in low-salinity, moderate-temperature environments but loses viscosity under harsher conditions. In contrast, HAPAM retains viscosity and stability even in high-salinity and high-temperature reservoirs, making it especially preferred for high-performance fracturing fluids. Its hydrophobic associations also improve shear resistance. PAM-PEI, on the other hand, forms durable gels suitable for high-temperature applications. However, strong breakers are required to degrade the synthetic polymers and minimize formation damage.

**Figure 12:**
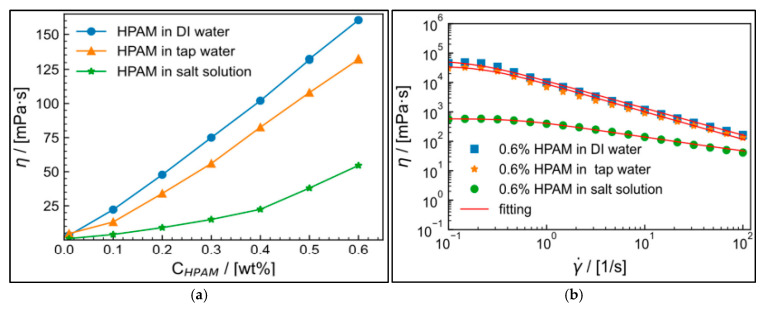
Conventional HPAM viscosity with different salinities (**a**) viscosity vs. HPAM concentration (**b**) Viscosity vs. shear rate [143].

Field trials, such as those involving the AquaPerm linear-gel system, have demonstrated the clear advantages of synthetic polymers, including cleaner breaks, lower pipe friction, high-rate pumping capability, and faster fluid recovery. While guar remains the default choice, synthetic alternatives are steadily proving their value in modern fracturing operations [84,141,142]. A recent study by Xin et al. (2023) [143] demonstrated that HPAM crosslinked with organic zirconium is a cost-effective and thermally stable option for fracturing fluid systems. The formulation maintained its viscosity at temperatures up to 200 °C when prepared with tap water and 180 °C in high-salinity conditions. Even under high temperature and shear stress, the HPAM–Zr system showed strong and stable performance. This was confirmed through rheological testing and cryo-SEM imaging, which revealed the formation of consistent viscoelastic gel structures. These findings show that synthetic polymers like HPAM can play a central role in designing fracturing fluids, especially for deep wells and high-salinity environments [143]. As shown in Figure 12, the rheological behavior of HPAM under different water qualities is compared. Figure 12a illustrates the apparent shear viscosity of HPAM in deionized water, tap water, and a mixed salt solution containing 1.5 × 10^4^ mg/L NaCl and 0.5 × 10^4^ mg/L CaCl_2_ at 30 °C and a shear rate of 100 s^−1^. Figure 12b presents the viscosity of 0.6 wt. % HPAM as a function of shear rate under the same fluid conditions, with solid lines representing the best fit using the Carreau model. Together, these figures highlight the significant impact of salinity on polymer viscosity and flow behavior [143].

## 7. Conclusions

This review highlights the critical role of polymers in hydraulic fracturing, focusing on their functional mechanisms, thermal stability, and interactions with additives under various reservoir conditions. Guar-based polymers, especially their derivatives like HPG and CMHPG, remain dominant due to their reliable performance, though concerns about residue and degradation persist. Modified cellulose polymers such as CMC offer low-residue alternatives with good thermal behavior but limited carrying capacity in high-shear environments. Xanthan gum, with excellent salinity tolerance and minimal residue, shows promise for saline reservoirs but is prone to thermal degradation and microbial activity. Synthetic polymers like PAM and HAPAM provide superior thermal and salt resistance and reduce formation damage, yet their environmental impact and cost must be considered. It is also suggested to inspect other sources of polymers, such as Arabic gum, which has huge potential to replace the Guar. As it is somehow cheap, it can achieve high viscosities and from a natural source. Cross-linking strategies and advanced breaker systems significantly influence gel behavior, proppant transport, and cleanup efficiency. The review also discusses the polymeric additives (FRs and SIs) used for other functions in fracturing fluids, their preparation, properties, role, and limitations. Future developments should emphasize environmentally sustainable, low-residue polymers, tailored formulations for produced water and seawater use, and biodegradable systems to minimize formation damage and environmental impact while optimizing hydraulic fracturing performance.

## Figures and Tables

**Table 1:** Polymer Classification by Source: Examples, Properties, and Applications.

Source	Polymer	General Properties	Applications
Plant-Based	Cellulose [42]	Linear polysaccharide	Hydraulic fracturing Textile industry (cotton, rayon)
		Insoluble, good interaction with water	Paper manufacturing
		High tensile strength	Biofuels
	Guar Derivatives [43]	Branched polysaccharide	Hydraulic fracturing Drilling fluids
		Hydrophilic	Food stabilizer
		Thickening and gelling properties	Pharmaceutical excipient
Microbial-Based	Xanthan Gum [44]	Branched polysaccharide	Food thickener
		Water-soluble	Oil drilling fluid stabilizer
		Viscosity-enhancing properties	Cosmetic emulsifier
Synthetic-Based	PAM [45]	Water-soluble synthetic polymer	Hydraulic fracturing, EOR, Water shut-off
		High viscosity in aqueous solutions	Water treatment and as an additive in fracturing operations
		Thermal stability	Soil conditioning
	PAA (Polyacrylic acid) [46]	Water-soluble	Hydraulic Fracturing, Water treatment
		High water absorption capacity	Superabsorbent materials
		pH-responsive	EOR

**Table 2:** Comparison between the most used polymers in hydraulic fracturing treatments.

Polymer	Gel Loading Viscosity	Preparation	Thermal Ranges	Salinity Tolerance	Performance, Residues and Cost
Guar	25–50 pptg 10–30 cP (linear) 500–2000 cP (cross-linked)	Grinding, Hydration, Cross-linking	Limited stability above 120 °C, improved with modifications	Low (<40,000 ppm, freshwater)	Cost-effective, high viscosity, struggles in high salinity and high temperature, 13% residues damage the formation.
HPG	25–50 pptg 20–50 cP (linear) Up to 1000 cP (cross-linked)	Remove insoluble oxidation, neutralization, washing, and drying	Stable up to 150 °C, retains viscosity better than guar	Moderate (up to 100,000 ppm)	Moderate cost, improved thermal stability, suitable for medium salinity, 3% residues, damage less compared to guar.
CMHPG	20–40 pptg 50–100 cP (linear) Up to 5000 cP (cross-linked)	Treating guar with propylene oxide and chloroacetic acid in the presence of NaOH, washing, drying	Stable up to 177 °C with stabilizers	High (up to 200,000 ppm)	Moderate-high cost, excellent for high salinity and high-temperature wells, minimal residue of 1%.
CMC	10–20 pptg 10–30 cP (linear) 500–1000 cP (cross-linked)	Alkalization, Etherification, Drying	Stable up to 100 °C, degrades at higher temperatures	High (up to brine levels >150,000 ppm)	Moderate cost, good proppant transport, but limited carrying capacity in high-shear environments. Low residue < 1%.
Xanthan Gum	1–3 pptg 20–50 cP (linear) 500–5000 cP (cross-linked)	Fermentation, Hydration, Cross-linking	Stable up to 120 °C, degrades above 75 °C	High (up to brine levels >150,000 ppm)	Moderate cost, poor thermal stability, excellent for saline and low-shear-rate conditions, low residue <1%.

**Table 3:** Approximate Prices of Common Fracturing Polymers (USD/kg).

Polymer	Approx. Price (USD/kg)	Notes
Guar gum	1.9 (US), 1.4 (China)	The most recent pricing information is obtained from (IMARC, 2025) [85]. Previously reported at $2/kg [61]
HPG	5.00	Slightly more expensive than raw guar gum
CMHPG	10.00	Highest among guar derivatives
CMC	5.00	Offers good performance at moderate cost
Xanthan gum	3.00	Moderate cost, common in high-salinity and low-shear environments
PHPA (partially hydrolyzed polyacrylamide)	0.17	Low-cost additive, used in WBDF and as support in fracturing fluids
PAM	2.44 (US), 0.83 (China)	Price varies by region; widely used in EOR and water treatment

## Data Availability

No new data were created or analyzed in this study.
